# Crystal structure of 3-chloro-1-methyl-5-nitro-1*H*-indazole

**DOI:** 10.1107/S2056989015018411

**Published:** 2015-10-10

**Authors:** Assoman Kouakou, El Mostapha Rakib, Mohamed Chigr, Mohamed Saadi, Lahcen El Ammari

**Affiliations:** a Laboratoire de Chimie Organique et Analytique, Université Sultan Moulay Slimane, Faculté des Sciences et Techniques, Béni-Mellal, BP 523, Morocco; b Laboratoire de Chimie du Solide Appliquée, Faculté des Sciences, Université Mohammed V, Avenue Ibn Battouta, BP 1014, Rabat, Morocco

**Keywords:** crystal structure, indazole derivative, Cl⋯O short contact

## Abstract

The mol­ecule of the title compound, C_8_H_6_ClN_3_O_2_, is built up from fused five- and six-membered rings connected to a chlorine atom and to nitro and methyl groups. The indazole system is essentially planar with the largest deviation from the mean plane being 0.007 (2) Å. No classical hydrogen bonds are observed in the structure. Two mol­ecules form a dimer organised by a symmetry centre *via* a close contact between a nitro-O atom and the chlorine atom [at 3.066 (2) Å this is shorter than the sum of their van der Waals radii].

## Related literature   

For biological activities such as as anti­microbial, anti­cancer, anti­inflammatory, anti­platelet and selective 5-HT6 antagonists of the title compound and derivatives, see: Schmidt *et al.* (2008 ▸); Shafakat Ali *et al.* (2012 ▸); Abbassi *et al.* (2014 ▸); Plescia *et al.* (2010 ▸); Lee *et al.* (2001 ▸); Liu *et al.* (2011 ▸).
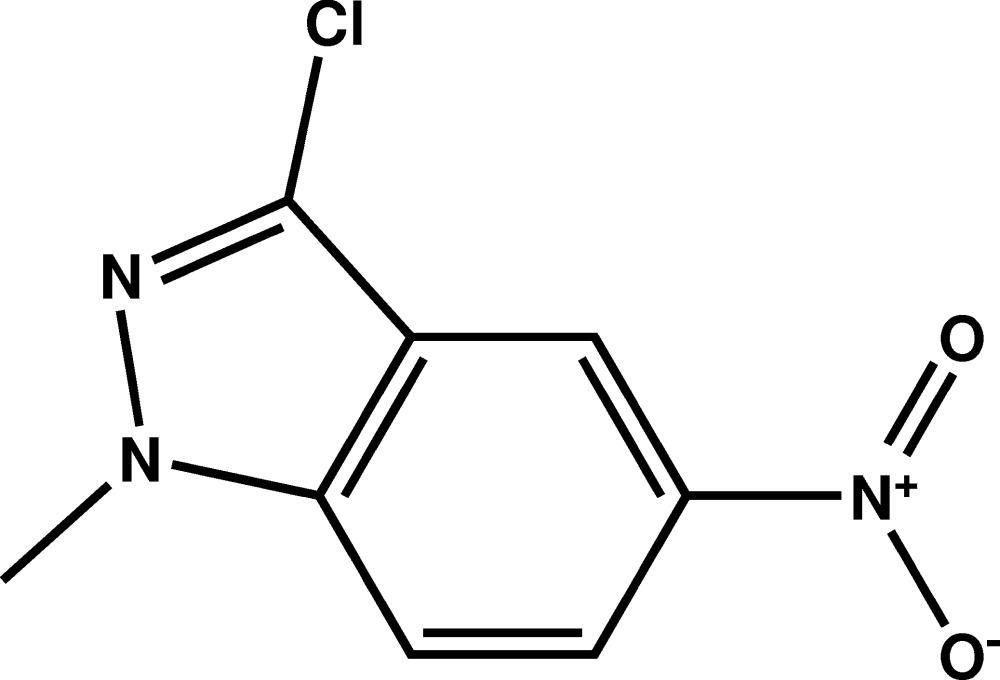


## Experimental   

### Crystal data  

C_8_H_6_ClN_3_O_2_
*M* *_r_* = 211.61
Monoclinic, 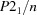

*a* = 3.8273 (2) Å
*b* = 14.678 (6) Å
*c* = 15.549 (6) Å
β = 96.130 (9)°
*V* = 868.5 (6) Å^3^
*Z* = 4
Mo *K*α radiation
μ = 0.41 mm^−1^
*T* = 296 K
0.31 × 0.27 × 0.21 mm

### Data collection  

Bruker X8 APEX Diffractometer
Absorption correction: multi-scan (*SADABS*; Bruker, 2009 ▸) *T*_min_ = 0.654, *T*_max_ = 0.747
19793 measured reflections
2243 independent reflections
1963 reflections with *I* > 2σ(*I*)
*R*_int_ = 0.028

### Refinement  

*R*[*F* ^2^ > 2σ(*F* ^2^)] = 0.043
*wR*(*F* ^2^) = 0.115
*S* = 1.10
2243 reflections
127 parameters
H-atom parameters constrained
Δρ_max_ = 0.36 e Å^−3^
Δρ_min_ = −0.27 e Å^−3^

### 

Data collection: *APEX2* (Bruker, 2009 ▸); cell refinement: *SAINT* (Bruker, 2009 ▸); data reduction: *SAINT*; program(s) used to solve structure: *SHELXS2013* (Sheldrick, 2008 ▸); program(s) used to refine structure: *SHELXL2013* (Sheldrick, 2015 ▸); molecular graphics: *ORTEP-3* (Burnett & Johnson, 1996 ▸; Farrugia, 2012 ▸); software used to prepare material for publication: *publCIF* (Westrip, 2010 ▸).

## Supplementary Material

Crystal structure: contains datablock(s) I. DOI: 10.1107/S2056989015018411/zp2019sup1.cif

Structure factors: contains datablock(s) I. DOI: 10.1107/S2056989015018411/zp2019Isup2.hkl

Supporting information file. DOI: 10.1107/S2056989015018411/zp2019Isup3.cml

**Fig. 1.**
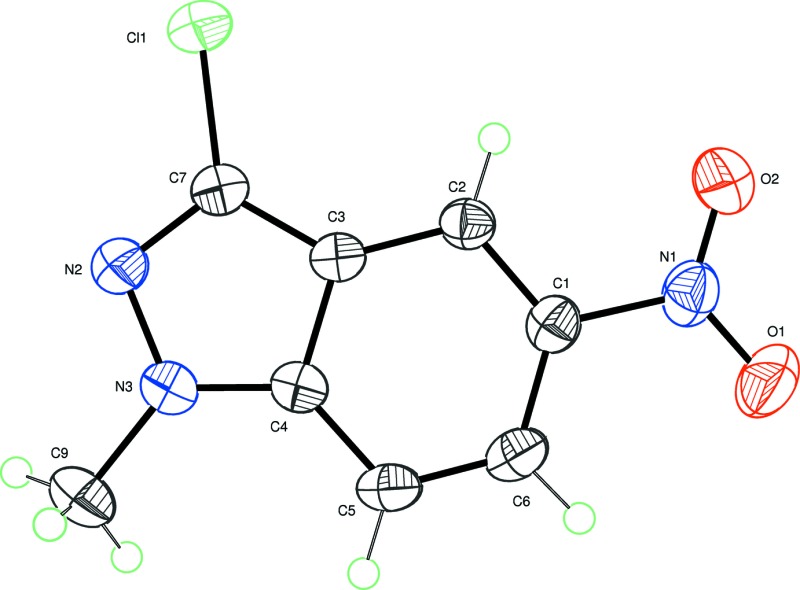
Plot of the molecule of the title compound with the atom-labelling scheme. Displacement ellipsoids are drawn at the 50% probability level. H atoms are represented as small circles.

. DOI: 10.1107/S2056989015018411/zp2019fig1.tif
Plot of the mol­ecule of the title compound with the atom-labelling scheme. Displacement ellipsoids are drawn at the 50% probability level. H atoms are represented as small circles.

CCDC reference: 1429148

Additional supporting information:  crystallographic information; 3D view; checkCIF report

## supplementary crystallographic information

### S1. Comment

Indazole derivatives are a versatile class of compounds that have found use in biology, catalysis, and medicinal chemistry. They exhibit a variety of biological activities such as anti-microbial, anti-cancer, anti-inflammatory, anti- platelet, and selective 5-HT6 antagonists (Schmidt *et al.*, 2008, Shafakat Ali *et al.*, 2012, Abbassi *et al.*, 2014, Plescia *et al.*, 2010, Lee *et al.*, 2001, Liu *et al.*, 2011).

The two fused five- and six-membered rings (N2N3 C1 to C7) part of the molecule are almost planar, with a maximum deviation of -0.007 (2) Å at C1 atom (Fig.1). The chlorine atom and the nitro group linked to the indazole ring are nearly coplanar with the largest deviation from the mean plane being -0.070 (2) Å at O1. No classic hydrogen bonds are observed in the structure.

### S2. Experimental

To a solution of 3-chloro-5-nitroindazole (6.13 mmol) in acetone (15 ml) was added potassium hydroxide (6.8 mmol). After 15 mn at 298 K, methyl iodide (12.26 mmol) was added dropwise. Upon disappearance of the starting material as indicated by TLC, the resulting mixture was evaporated. The crude material was dissolved with EtOAc (50 ml), washed with water and brine, dried over MgSO_4_ and the solvent was evaporated *in vacuo*. The resulting residue was purified by column chromatography (EtOAc/hexane 2/8). The title compound was recrystallized from ethanol at room temperature giving colourless crystals (m.p. 471 K, yield: 70%).

### S3. Refinement

H atoms were located in a difference map and treated as riding with C–H = 0.96 Å and C–H = 0.93 Å for methyl and aromatic, respectively. All hydrogen with *U*_iso_(H) = 1.5 *U*_eq_ for methyl and *U*_iso_(H) = 1.2 *U*_eq_ for aromatic.

### Crystal data

**Table:**

C_8_H_6_ClN_3_O_2_	*D*_x_ = 1.618 Mg m^−^^3^
*M**_r_* = 211.61	Melting point: 471 K
Monoclinic, *P*2_1_/*n*	Mo *K*α radiation, λ = 0.71073 Å
*a* = 3.8273 (2) Å	Cell parameters from 2243 reflections
*b* = 14.678 (6) Å	θ = 2.6–28.7°
*c* = 15.549 (6) Å	µ = 0.41 mm^−^^1^
β = 96.130 (9)°	*T* = 296 K
*V* = 868.5 (6) Å^3^	Block, colourless
*Z* = 4	0.31 × 0.27 × 0.21 mm
*F*(000) = 432	

### Data collection

**Table:**

Bruker X8 APEX Diffractometer	2243 independent reflections
Radiation source: fine-focus sealed tube	1963 reflections with *I* > 2σ(*I*)
Graphite monochromator	*R*_int_ = 0.028
φ and ω scans	θ_max_ = 28.7°, θ_min_ = 2.6°
Absorption correction: multi-scan (*SADABS*; Bruker, 2009)	*h* = −5→5
*T*_min_ = 0.654, *T*_max_ = 0.747	*k* = −19→19
19793 measured reflections	*l* = −20→20

### Refinement

**Table:**

Refinement on *F*^2^	0 restraints
Least-squares matrix: full	Hydrogen site location: inferred from neighbouring sites
*R*[*F*^2^ > 2σ(*F*^2^)] = 0.043	H-atom parameters constrained
*wR*(*F*^2^) = 0.115	*w* = 1/[σ^2^(*F*_o_^2^) + (0.0484*P*)^2^ + 0.5276*P*] where *P* = (*F*_o_^2^ + 2*F*_c_^2^)/3
*S* = 1.10	(Δ/σ)_max_ < 0.001
2243 reflections	Δρ_max_ = 0.36 e Å^−^^3^
127 parameters	Δρ_min_ = −0.27 e Å^−^^3^

### Special details

**Table:**

Geometry. All e.s.d.'s (except the e.s.d. in the dihedral angle between two l.s. planes) are estimated using the full covariance matrix. The cell e.s.d.'s are taken into account individually in the estimation of e.s.d.'s in distances, angles and torsion angles; correlations between e.s.d.'s in cell parameters are only used when they are defined by crystal symmetry. An approximate (isotropic) treatment of cell e.s.d.'s is used for estimating e.s.d.'s involving l.s. planes.

### Fractional atomic coordinates and isotropic or equivalent isotropic displacement parameters (Å^2^)

**Table:**

	*x*	*y*	*z*	*U*_iso_*/*U*_eq_	
Cl1	0.39641 (14)	0.41233 (3)	0.10469 (3)	0.04683 (17)	
N3	0.7340 (4)	0.56202 (10)	0.29223 (9)	0.0345 (3)	
N2	0.6938 (4)	0.48003 (10)	0.25083 (10)	0.0358 (3)	
N1	0.0460 (5)	0.79442 (11)	0.05310 (10)	0.0416 (4)	
O2	−0.0907 (5)	0.75957 (11)	−0.01323 (11)	0.0671 (5)	
O1	0.0360 (6)	0.87596 (11)	0.06625 (11)	0.0669 (5)	
C3	0.4260 (4)	0.59119 (11)	0.16618 (10)	0.0283 (3)	
C4	0.5778 (4)	0.63052 (11)	0.24420 (10)	0.0299 (3)	
C2	0.2473 (4)	0.64453 (11)	0.10193 (10)	0.0301 (3)	
H2	0.1459	0.6196	0.0502	0.036*	
C1	0.2289 (5)	0.73599 (12)	0.11912 (11)	0.0325 (3)	
C7	0.5114 (4)	0.49799 (12)	0.17658 (11)	0.0320 (3)	
C5	0.5521 (5)	0.72408 (12)	0.26022 (11)	0.0373 (4)	
H5	0.6498	0.7497	0.3120	0.045*	
C6	0.3773 (5)	0.77622 (12)	0.19662 (12)	0.0381 (4)	
H6	0.3564	0.8387	0.2047	0.046*	
C9	0.9194 (5)	0.56629 (15)	0.37845 (12)	0.0442 (5)	
H9A	1.0018	0.5066	0.3957	0.066*	
H9B	1.1160	0.6069	0.3785	0.066*	
H9C	0.7634	0.5882	0.4182	0.066*	

### Atomic displacement parameters (Å^2^)

**Table:**

	*U*^11^	*U*^22^	*U*^33^	*U*^12^	*U*^13^	*U*^23^
Cl1	0.0591 (3)	0.0334 (2)	0.0457 (3)	0.00152 (19)	−0.0049 (2)	−0.01074 (18)
N3	0.0347 (8)	0.0374 (8)	0.0308 (7)	−0.0012 (6)	0.0006 (6)	−0.0012 (6)
N2	0.0367 (8)	0.0345 (7)	0.0360 (7)	0.0011 (6)	0.0033 (6)	−0.0012 (6)
N1	0.0497 (9)	0.0357 (8)	0.0405 (8)	0.0048 (7)	0.0095 (7)	0.0062 (6)
O2	0.0955 (14)	0.0476 (9)	0.0513 (9)	0.0052 (9)	−0.0246 (9)	0.0045 (7)
O1	0.1054 (15)	0.0352 (8)	0.0589 (10)	0.0147 (9)	0.0037 (9)	0.0054 (7)
C3	0.0266 (8)	0.0304 (8)	0.0285 (7)	−0.0028 (6)	0.0056 (6)	−0.0032 (6)
C4	0.0270 (8)	0.0348 (8)	0.0284 (7)	−0.0043 (6)	0.0051 (6)	−0.0020 (6)
C2	0.0310 (8)	0.0321 (8)	0.0276 (7)	−0.0024 (6)	0.0048 (6)	−0.0014 (6)
C1	0.0342 (9)	0.0315 (8)	0.0328 (8)	−0.0001 (7)	0.0084 (7)	0.0029 (6)
C7	0.0317 (8)	0.0314 (8)	0.0331 (8)	−0.0018 (6)	0.0047 (6)	−0.0039 (6)
C5	0.0430 (10)	0.0359 (9)	0.0330 (8)	−0.0069 (7)	0.0044 (7)	−0.0093 (7)
C6	0.0463 (10)	0.0283 (8)	0.0407 (9)	−0.0028 (7)	0.0100 (8)	−0.0058 (7)
C9	0.0422 (10)	0.0559 (12)	0.0326 (9)	−0.0021 (9)	−0.0055 (8)	−0.0008 (8)

### Geometric parameters (Å, º)

**Table:**

Cl1—C7	1.7086 (18)	C4—C5	1.401 (2)
N3—C4	1.353 (2)	C2—C1	1.372 (2)
N3—N2	1.366 (2)	C2—H2	0.9300
N3—C9	1.450 (2)	C1—C6	1.406 (3)
N2—C7	1.311 (2)	C5—C6	1.367 (3)
N1—O1	1.215 (2)	C5—H5	0.9300
N1—O2	1.218 (2)	C6—H6	0.9300
N1—C1	1.458 (2)	C9—H9A	0.9600
C3—C2	1.390 (2)	C9—H9B	0.9600
C3—C4	1.411 (2)	C9—H9C	0.9600
C3—C7	1.412 (2)		
			
C4—N3—N2	111.95 (14)	C2—C1—N1	117.97 (16)
C4—N3—C9	128.47 (16)	C6—C1—N1	118.43 (16)
N2—N3—C9	119.56 (16)	N2—C7—C3	113.01 (15)
C7—N2—N3	105.10 (14)	N2—C7—Cl1	120.27 (14)
O1—N1—O2	122.53 (18)	C3—C7—Cl1	126.72 (13)
O1—N1—C1	118.81 (17)	C6—C5—C4	117.27 (16)
O2—N1—C1	118.66 (16)	C6—C5—H5	121.4
C2—C3—C4	120.87 (15)	C4—C5—H5	121.4
C2—C3—C7	135.89 (15)	C5—C6—C1	120.43 (16)
C4—C3—C7	103.23 (14)	C5—C6—H6	119.8
N3—C4—C5	131.73 (16)	C1—C6—H6	119.8
N3—C4—C3	106.71 (15)	N3—C9—H9A	109.5
C5—C4—C3	121.56 (16)	N3—C9—H9B	109.5
C1—C2—C3	116.26 (15)	H9A—C9—H9B	109.5
C1—C2—H2	121.9	N3—C9—H9C	109.5
C3—C2—H2	121.9	H9A—C9—H9C	109.5
C2—C1—C6	123.60 (16)	H9B—C9—H9C	109.5
