# Supplementary figures and images for: Crystal structure of 3-chloro-1-methyl-5-nitro-1H-indazole
Source: Acta Crystallogr E Crystallogr Commun. 2015 Oct 10;71(Pt 11):o834–5. doi: 10.1107/S2056989015018411 (PMC4645045; doi:10.1107/S2056989015018411)

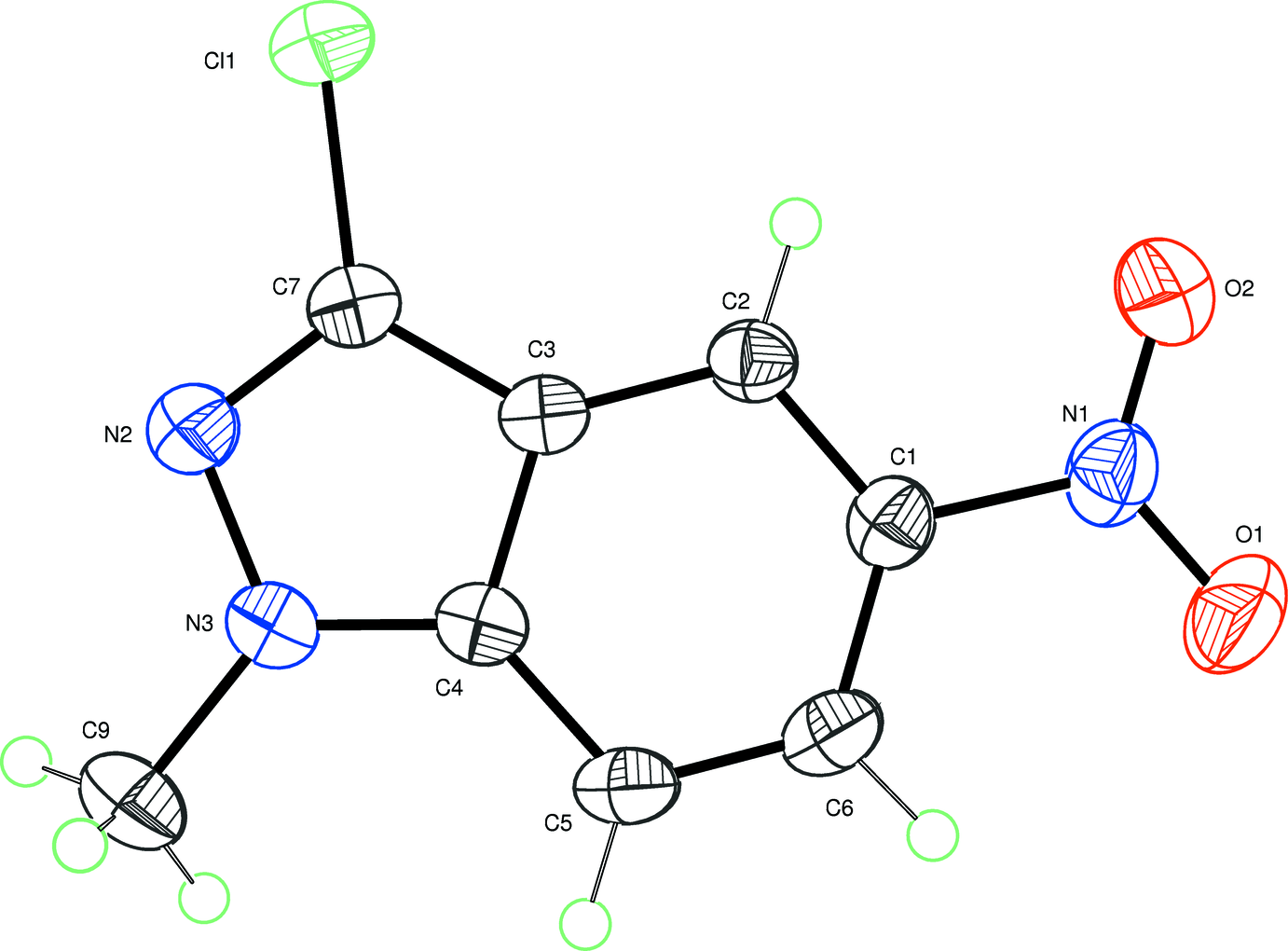

Supplement: Supplementary file 4 [file e-71-0o834-fig1.tif]
